# Color standardization and optimization in Whole Slide Imaging

**DOI:** 10.1186/1746-1596-6-S1-S15

**Published:** 2011-03-30

**Authors:** Yukako Yagi

**Affiliations:** 1 Department of Pathology, Massachusetts General Hospital and Harvard Medical School 101 Merrimac St. Suite 820, Boston, MA 02114, USA

## Abstract

**Introduction:**

Standardization and validation of the color displayed by digital slides is an important aspect of digital pathology implementation. While the most common reason for color variation is the variance in the protocols and practices in the histology lab, the color displayed can also be affected by variation in capture parameters (for example, illumination and filters), image processing and display factors in the digital systems themselves.

**Method:**

We have been developing techniques for color validation and optimization along two paths. The first was based on two standard slides that are scanned and displayed by the imaging system in question. In this approach, one slide is embedded with nine filters with colors selected especially for H&E stained slides (looking like tiny Macbeth color chart); the specific color of the nine filters were determined in our previous study and modified for whole slide imaging (WSI). The other slide is an H&E stained mouse embryo. Both of these slides were scanned and the displayed images were compared to a standard. The second approach was based on our previous multispectral imaging research.

**Discussion:**

As a first step, the two slide method (above) was used to identify inaccurate display of color and its cause, and to understand the importance of accurate color in digital pathology. We have also improved the multispectral-based algorithm for more consistent results in stain standardization. In near future, the results of the two slide and multispectral techniques can be combined and will be widely available.

We have been conducting a series of researches and developing projects to improve image quality to establish Image Quality Standardization. This paper discusses one of most important aspects of image quality – color.

## Background

Technologies in WSI have been improving for the last decade [1]. A variety of scanners are now available with faster scanning speed and higher image quality [2]. The usages of WSI are varied such as for developing decision support system, image analysis, education, conference and remote diagnosis. However, there are still many issues we must solve before implementation in the clinical environment such as the system stability, consistency of image quality, etc. The issues are becoming even more serious as WSI is becoming popular and used as a part of clinical practice. One of the most important issues in WSI is the color. Standardization and validation of the color of digital slides on the display is an important aspect of digital pathology implementation. While the most common reason for color variation is the variance in the protocols and practices in the histology lab, the color displayed can also be affected by variation in capture parameters (for example, illumination and filters), image processing and display factors in the digital systems themselves. All processes are very important and influence each other. Five major reasons of color variation are thickness of specimen, staining, scanner, viewer and display. To realize that the color we are looking at is not optimized or standardized is the first step towards standardization. Because no one who is involved in the process, i.e. between making a slide and displaying it, looks at the slide color at each step. Most of them are only responsible for 1-2 processes. For example, a histology technician looks at the physical stain dyes and a stained slides only; the person scanning a slide checks the stained slide and scanned image, and a reviewer looks at the images on her/his display in remote server. The advantage of WSI is to minimize the physical distance between the slide and the reviewer and also among reviewers. It is difficult to know whether the appropriate color of the WSI is displayed at the reviewers’ end, or even at a local display station. It is a huge challenge to standardize the color in the entire process (staining to displaying the scanned slides), for anyone at anywhere.

Five major causes of color issues are following.

### Thickness of specimen

**Figure 1:**
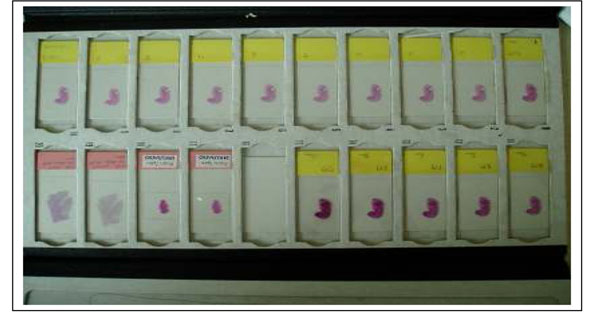


**Figure 2:**
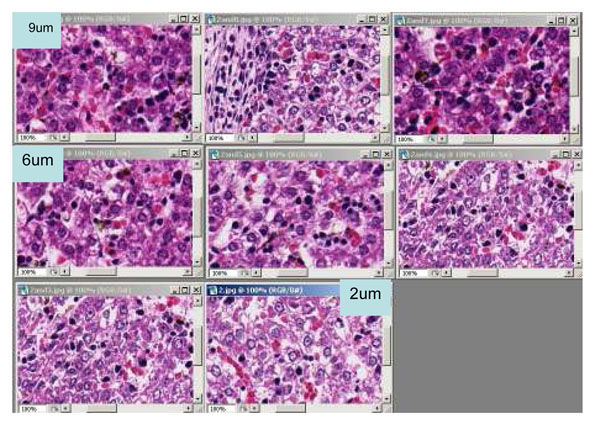


Generally the thickness of the specimen in the USA is 4-7 um. However this number is not measured thickness, it is targeted and expected thickness. The thickness of across the tissue section is often not uniform especially when the tissue size is relatively large such as surgical resection samples. An automated staining machine is used for H&E stain at major histology lab in most countries. Figure 1 shows the difference of stain dyes absorbance by tissue thickness. The tissues were sectioned by an automated sectioning machine to have good consistency in thickness and quality of tissue. Figure 2 shows digitized images of slides in Figure 1. Thicker tissue slides show darker and unclear details of tissue. Thinner tissue slides show clearer details with lighter color. Thus, the thickness of specimen influences the color appearance of stained slide and scanned image.

### Staining

**Figure 3:**
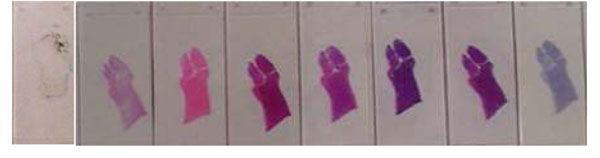


The appearance of H&E stained slide varies between laboratories or institutions. So that there is more confusion from viewing digitized slides (WSI) compared to observing the slides under a microscope because the actual stained slides can not be seen. Figure 3 shows the color variations of H&E stained slide. It has to be standardized or adjusted to the preferred color of each pathologist. Another critical effect of staining is the image analysis results. To have consistent image analysis results, the appearance of staining should be standardized.

### Scanner and scanning process

**Figure 4:**
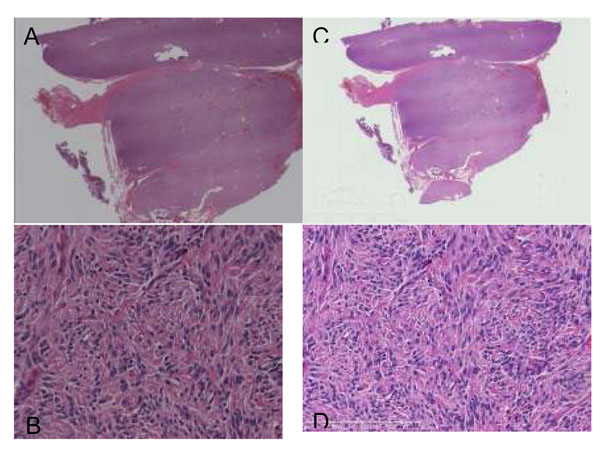


Scanner and Scanning process also produce differences of color appearance. A scanner is a combination of many components such as optics, image acquisition device and image acquisition algorithm; this is the most complicated part in the implementation of color standardization. Figure 4 shows sample images highlighting the color differences between scanners. Both images in Figure 4 were displayed on the same display.

### Viewer

**Figure 5:**
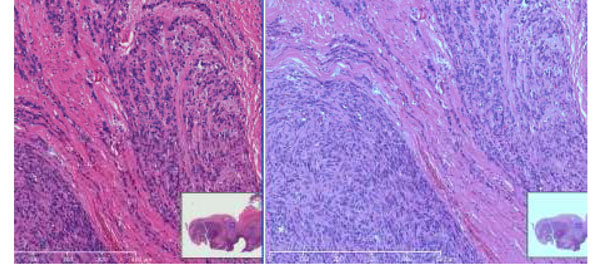


Some of viewers can show multiple WSI produced by other scanners and it is very useful functions. However, the image quality and color appearance often differ with different viewers. Figure 5 shows an example of the difference by the viewer. Original image and display are same.

### Display

Display is another cause of color variations. Recently there is a variety of display types having varying size and resolution, and each has many settings to change. There are also many choices of display cards. The matching between display card, display type and computer specification is important to see the original imaging data best. However, to attain the best performance of each device is often difficult. Most of the time, we use it inefficiently without noticing.

**Figure 6:**
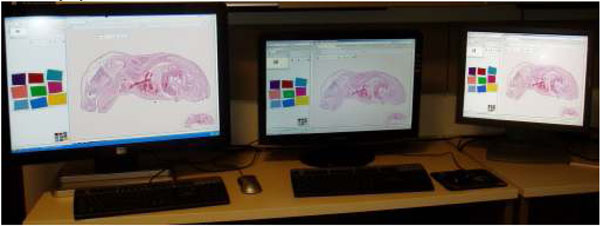


Figure 6 shows the example of the differences between three displays. Original image and computer connected to the 3 displays are the same.

Users including pathologists and engineers at the imaging facility [like us] have limited control of the thickness of the specimen, staining process, the color of an image which a scanner produced and the viewer. However, if it is possible to know how and how much is the acquired inaccuracy then we could optimize the displayed images. The staining condition can be controlled and standardized using spectral information. This is discussed in our other papers [3]. Therefore, the aims of this study were to understand the variation of the color in WSI environment and to establish a simple protocol to improve the color towards Color standardization.

## Materials and methods

We have conducted two experiments, 1. Display evaluation and 2. Establish a protocol towards standardization.

### Display evaluation

**Figure 7:**
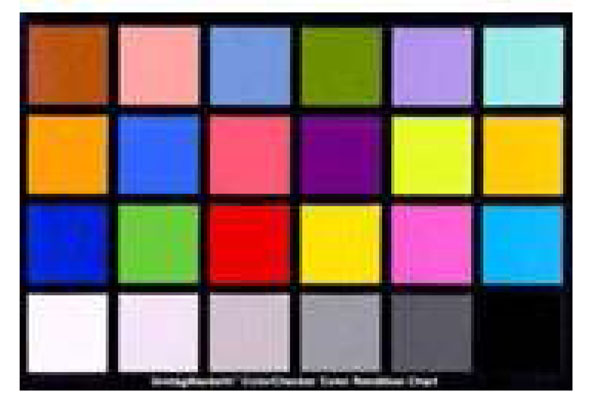


In color-related fields, a color chart is a physical arrangement of standardized color samples, used for color comparisons and measurements such as in checking the color reproduction of an imaging system. Color charts are used to calibrate and to profile graphic devices, such as digital cameras and scanners. Therefore standardized IT8 targets are made. One of most common charts is the Macbeth color chart in Figure 7.

**Figure 8:**
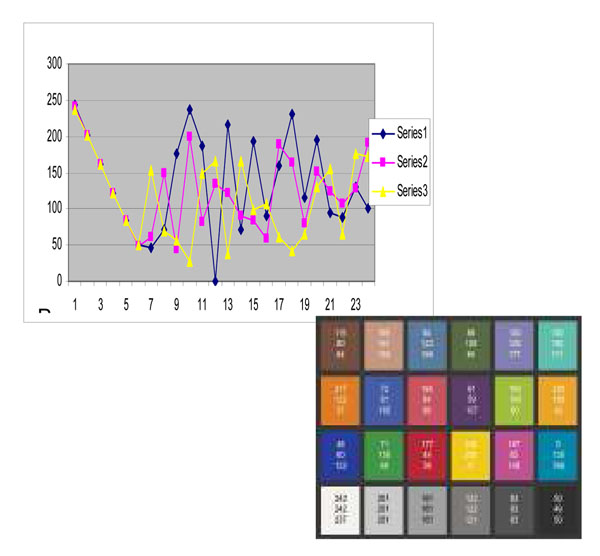


We have adapted Macbeth color chart to investigate the current condition of the displays in the department. RGB values of each patch are shown in Figure 8. Macbeth chart (1280x1024 pixel) was prepared in the website.

Our Department has 2 major models as standard for our clinical use. We have randomly selected 23 of one of the standard displays, HP Compaq LA 1750.

We confirmed that all setting of display setting in the PC and display itself were same for all 23 displays. The age of each display was undetermined. Display analyzer, Anaheim Scientific was used to measure RGB/HSL value of each patch. If an owner of each display desired to calibrate after the measurement, we calibrated using Monitor Calibration tool, Eye One Display LT, X-Rite.

### Towards to standardization and optimization

**Figure 9:**
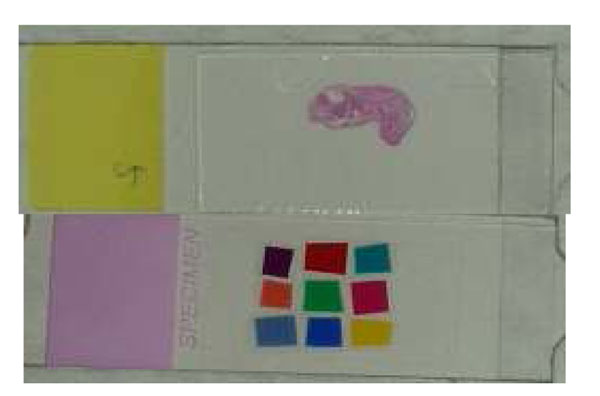


Figure 9 shows a set of calibration slides, one is a color chart we developed for WSI and an H&E stained slide of a mouse embryo.

#### In house color chart

9 Color filters for color chart slides were selected for histology stained especially work for H&E stain best [3]. Initially, the slide was made for a microscope based imaging system and it fits within 4x objective lens’s field of view and made for accurate color reproduction for pathology imaging especially for telepathology.

The scanning area of common WSI scanner is the entire glass slide or 1x1.5 inch^2^ so that it is relatively easier to make the color chart slide for WSI than for microscopes.

Because the current slides are hand made and we recognized some dusts and finger prints between color charts and cover tape on the slide, we measured the spectral information of each filter by spectrometer. All data should be recorded and stored in the color management database.

#### Mouse embryo H&E slide

A simple protocol which anyone can perform comfortably is important and necessary to be widely accepted. After the color calibration by the color chart, pathologist can confirm the color again with mouse embryo H&E stained WSI. The mouse embryo tissue sample contains most of organ system even though each organ has not grown enough yet. 100 slides from one block were sectioned by the automated sectioning machine AS200S, Kurabo LTD, Japan with 3um thickness and stained with H&E by the automated H&E staining machine at once. All 100 slides were scanned at the lab and stored in the color management database.

## Results

### Display evaluation

**Figure 10:**
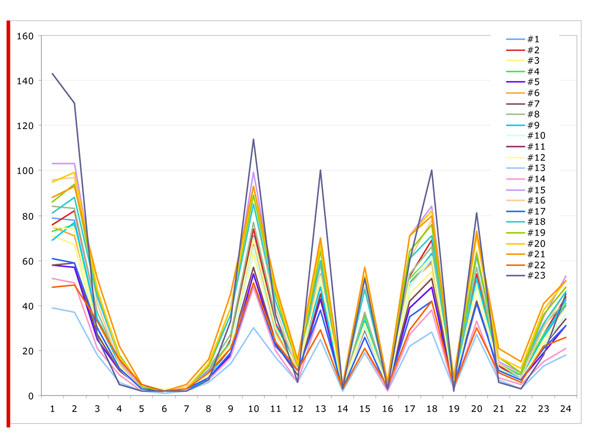


**Figure 11:**
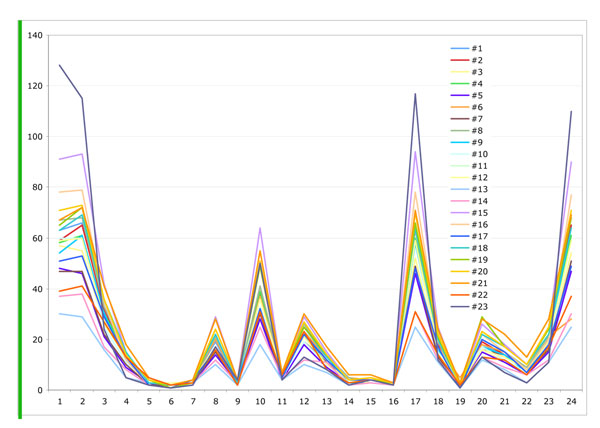


**Figure 12:**
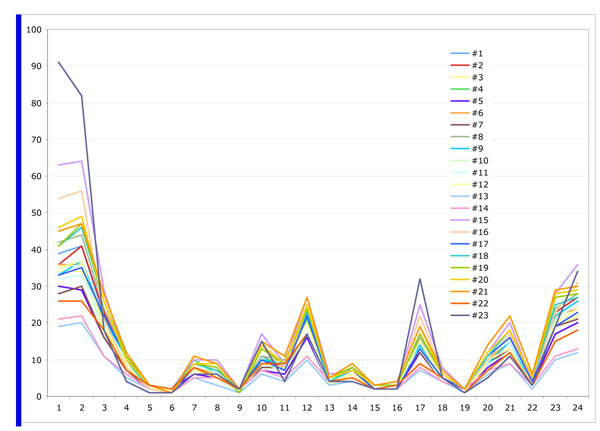


Figure 10, 11,12 show the measurement results of randomly selected 23 displays in the department. Figure 10 shows the red value, Figure 11 shows the green value and Figure 12 shows the blue value of each 24 Macbeth color cart. None of them had exact same value with any of other displays although all displays were the same model. All three graphs of most of the displays showed lower value for white patch, No 1. (than gray) No. 2 and 3. It means more than 20 displays could not show white accurately and white patches in some of display are even darker than gray patches (No2.).

**Figure 13:**
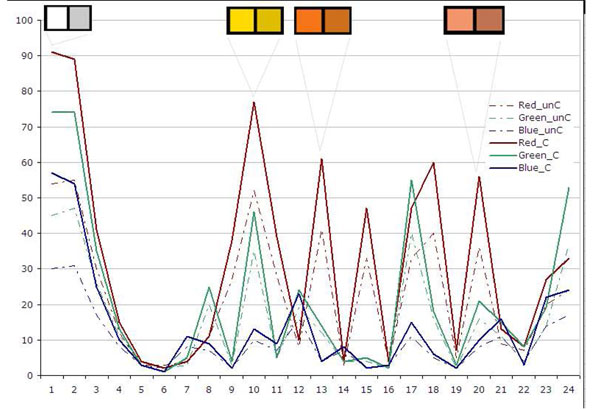


From the graphs, we can see that our displays were not well optimized and calibrated. Also some of the colors were shifted to other colors. We have calibrated some of the displays after the initial measurement. Figure 13 shows an example of one display’s difference of RGB values before and after the calibration. The displays shows more contrast and collect color such as white. Figure 13 shows how much each color was differentiated. The calibration tool we used in this study is not ideal calibration tool. However, its cost is reasonable and help improved the color condition of the displays.

### In house color chart

As mentioned earlier, we found that our display is not standardized and calibrated ideally. Using the color chart we developed, we performed experiments to check the color condition of the scanner and the display. Macbecth color chart is very useful chart to check the color representation. However, measuring the display often for each 24 patches is not a very realistic method to implement the color standardization in the entire department. Simpler method is required.

### Scanner

**Figure 14:**
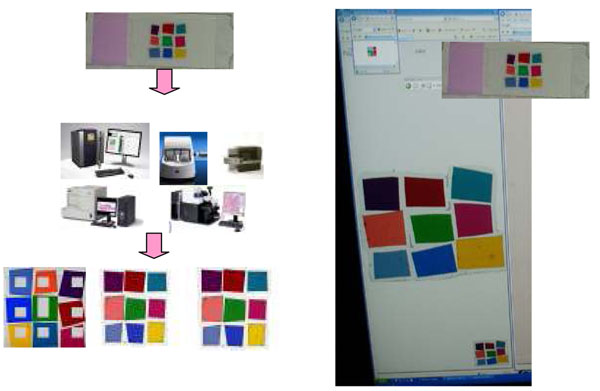


Figure 14 shows the protocol to check the color condition of a scanner using the calibration slide. First, the calibration slide is scanned by a scanner. Second, a user goes to the calibration slide website to see the original slide on the display. The difference of each color patch is the color shift that produced by a scanner. In this paper, we do not discuss how to adjust the color. By measuring each color in the calibration slide and web site, we can calculate the shift of each color.

**Figure 15:**
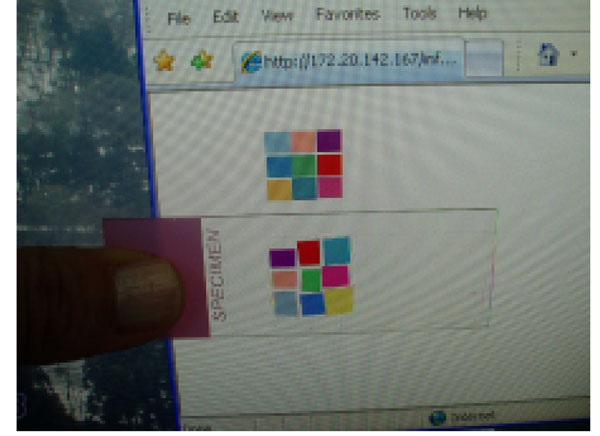


Figure 15 shows the display color evaluation protocol on how to know whether the color on our displays is acceptable or not. Since the color calibration slide is in-house with very low cost, every user can keep it to use at anytime. First, a user looks at the calibration web site then checks if all the 9 colors displayed at his/her monitors are differentiated or not. From our experience, some of the patches showed very similar color and it becomes difficult to identify which patch on the display is which color patch on the slide. We recommend the calibration of the display when this occurs. If each color is differentiated, the next step is to compare the color of each patch on the display with the actual slide. In this paper, we don’t discuss how to standardize the display. The purpose is to know how much the display can produce colors correctly.

**Figure 16:**
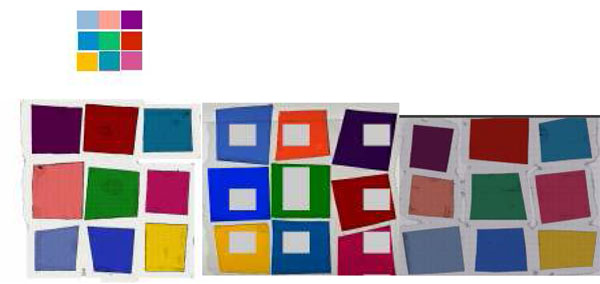


Figure 16 shows the scanned results of the calibration slide by 3 scanners. 5 scanners were tested. None of the scanner could produce exactly the same colors although some of scanners have their own calibration material or integrated a software color calibration function in the system.

**Figure 17:**
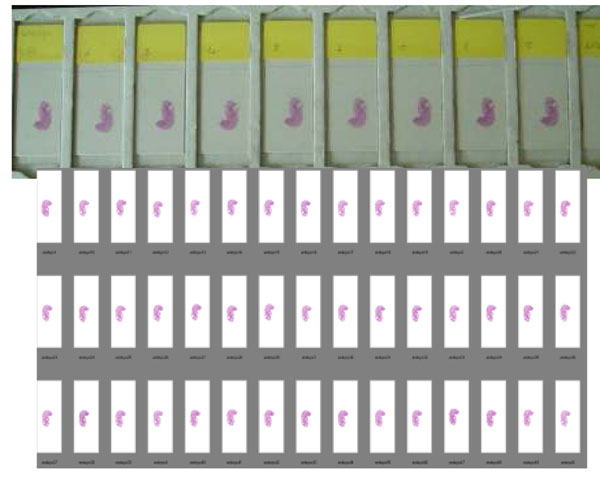


Figure 17 shows the mouse embryo slides which were sectioned using an automated sectioning machine and stained with H&E by an automated stainer at the same time.

All slides have very close H&E stained appearance and all slides were scanned at our lab as reference. The purpose of these slides is not only for the color optimization.

Some pathologists are not comfortable to use only the color patch slide to check the color reproduction even though the colors were selected for major histology stained slides. To make a pathologist more comfortable, we use the slides (H&E stained mouse embryo slides) as part of the color calibration slides. Using the slide scanned image pathologist can look at each component of each organ if desired. Given the slides, it is easier to understand the color shift by time (continuous observation of color condition of a system) than color patch.

Although it is more difficult to identify which color has shifted and how much is the shift compared to the color calibration slide, the overall color reproduction is easier to evaluate with this type of slide.

## Discussion

Although the methods described in this paper are not ideal and there are still some missing components, but through the conducted experiments, pathologists become more sensitive to the color of the image and the color of displays than before. We have been developing the protocol and algorithm for standard slides and multispectral technologies for color standardization in relation to WSI such as color standardization for entire process and image quality standardization as well. Staining standardization was not discussed in this paper (we have been having a promising data for H&E standardization). In future, we will integrate these technologies into the WSI system.

### Support

One of the great benefits of WSI is to support developing counties or rural area of pathologists. WSI are being used for education and remote diagnosis.

We will need some kind of method to support the entire world. We have given some of our calibration slides to some of the developing countries so that we can evaluate their image/color quality and also monitor them over time. One of difficulties we experienced was seeing their WSI. Some institutions which have been using the WSI do not have the web server to host their images. To manage and view their images were not so easy although not impossible. Recently we use public web site to share the calibration images with user name password controls.

### Standard

Last several years, DICOM Group 26 has been working on the standardization of WSI file format, data structure and metadata. It is very important activities to promote WSI. At the same time, the standardization of image quality, color reproduction, quality of image analysis result, and etc is important to use WSI safely. I hope that the international or national organization like International Telecommunication Union or National Institute of Standards and Technology would support us to accomplish it.

### Color

We are not certain if color and image quality are very important to keep high quality WSI or not. The answer would be different on a case by case basis. In the near future it is possible that WSI would replace microscope even for clinical applications. Even small error in color reproduction, image reconstruction, or analysis results could cause some kind of misinterpretation of image. To prevent any errors in imaging, “color” standardization is one of the most important aspects.

## Conclusion

The Macbeth color chart study was very useful to understand the displays condition across our entire department. A pair of calibration slides helps us to understand the color problem, and using the calibration slides we were able to improve the color of the displays by ourselves.

## Competing interests

The authors declare that they have no competing interests.
